# Validation of the Distress Thermometer in patients with advanced cancer receiving specialist palliative care in a hospice setting

**DOI:** 10.1177/0269216320954339

**Published:** 2020-09-11

**Authors:** Lisa Graham-Wisener, Martin Dempster, Aaroon Sadler, Luke McCann, Noleen K McCorry

**Affiliations:** 1 Centre for Improving Health-Related Quality of Life, School of Psychology, Queen’s University Belfast, Belfast, UK; 2 Marie Curie Hospice Belfast, Belfast, UK; 3 South Eastern Health and Social Care Trust, Ulster Hospital, Dundonald, Downpatrick, UK; 4 Centre of Excellence in Public Health, Queen’s University Belfast, Belfast, UK

**Keywords:** Palliative care, terminal care, anxiety, depression, validation studies, distress thermometer, advanced cancer, distress

## Abstract

**Background:**

Ongoing assessment of psychological reaction to illness in palliative and end of life care settings is recommended, yet validated tools are not routinely used in clinical practice. The Distress Thermometer is a short screening tool developed for use in oncology, to detect individuals who would benefit from further psychological assessment. However the optimal cut-off to detect indicative psychological morbidity in patients with advanced cancer receiving specialist palliative care is unclear.

**Aim:**

To provide the first validation of the Distress Thermometer in an advanced cancer population receiving specialist palliative care in a UK hospice setting.

**Design:**

Receiver Operating Characteristics analysis was used to compare the sensitivity and specificity of cut-offs indicative of psychological morbidity on the Distress Thermometer in comparison to the Hospital Anxiety and Depression Scale.

**Setting/Participants:**

Data were derived from 202 patients with advanced cancer who were approached on admission to inpatient or day hospice care, with 139 patients providing complete data on both measures.

**Results:**

The area under the curve was optimal using a Distress Thermometer cut-off score of ⩾6 for total distress and for anxiety, and a cut-off score of ⩾4 optimal when screening for depression.

**Conclusions:**

The Distress Thermometer is a valid, accurate screening tool to be used in advanced cancer but with caution in relation to the lack of specificity. With little variation between the area under the curve scores, arguably a Distress Thermometer cut-off score of ⩾5 is most appropriate in screening for all types of psychological morbidity if sensitivity is to be prioritised.

**What is already known about the topic?**

- There is variation in the recommended Distress Thermometer cut-off scores for detecting indicative psychological morbidity, with need for revalidation when introducing the Distress Thermometer into new clinical populations, settings and cultures.
- There is a lack of consistency in the cut-off scores proposed by the limited number of Distress Thermometer validation studies within a palliative and end of life care setting, and no evidence of the optimal cut-off score for implementation of the Distress Thermometer with patients with advanced cancer in inpatient and day hospice settings

**What this paper adds**

- A cut-off score of ⩾6 is optimal when screening for total distress and for anxiety, and a Distress Thermometer cut-off score of ⩾4 is optimal when screening for depression in patients with advanced cancer receiving specialist palliative care in an inpatient/day hospice setting.
- With little variation between the area under the curve scores, arguably a Distress Thermometer cut-off score of ⩾5 is most appropriate in screening for all types of psychological morbidity if sensitivity is to be prioritised.
- Accuracy of the Distress Thermometer in screening for indicative psychological morbidity is fair to good in relation to sensitivity, but poor in relation to specificity with a number of false positives.

**Implications for practice, theory or policy**

- The Distress Thermometer is an accurate, valid screening tool for depression, anxiety and distress, and may be implemented in routine clinical practice to identify patients with advanced cancer receiving specialist palliative care in an inpatient/day hospice setting who would benefit from further psychological assessment.

## Background

Psychological distress is defined as “a multi-factorial unpleasant emotional experience” which extends along a continuum, including a range of psychological morbidity from normal feelings of vulnerability to mood disorders including depression and anxiety disorder.^1^ Heightened levels of psychological morbidity are reported in patients with advanced cancer receiving palliative and end of life care, with pooled prevalence of major depression at 14.3% and anxiety disorder at 9.8% according to Diagnostic and Statistical Manual of Mental Disorders or International Classification of Diseases definitions of psychological morbidity.^2^ Untreated psychological morbidity is associated with increased physical symptom burden,^3,4^ more challenging symptom management,^5^ lack of acceptance of prognostic information,^6^ a reduction in global health-related quality of life,^7^ and may also be an independent prognostic indicator.^8^ Importantly, when identified, there is evidence that psychological morbidity is amenable to change.^9^ A systematic review recently established the effectiveness of brief psychosocial interventions (median of *n* = 2 sessions) on emotional distress among patients receiving palliative care.^10^

Evidence of complex need differentiates patients with advanced cancer who should be cared for by specialist palliative care services from those for whom non-specialist care is most appropriate.^11^ Although there is a lack of certainty over how complexity is defined, complex emotional symptoms have been identified as a criteria by an international consensus^12^ and healthcare professionals.^13^ Clinical guidelines and quality indicators for specialist palliative care^14,15^ recommend ongoing assessment of psychological reaction to illness with validated assessment tools. There is however indication that validated tools are not frequently used in specialist palliative care settings,^16,17^ with psychological morbidity potentially underreported and undertreated.^18^

Ultra-short screening tools ( < 5 items) are attractive to clinicians because of their ease of use,^18^ followed by a more lengthy assessment undertaken only for patients reporting clinically significant scores. The Distress Thermometer^19^ is a single-item, 11-point visual analogue scale, with respondents indicating how distressed they have felt over the past week (from “No Distress” to “Extreme Distress”). In their guidelines the National Comprehensive Cancer Network suggest a cut-off score of ⩾5 in oncology samples as indicative of significant distress requiring additional assessment and treatment.^20^ However, a meta-analysis of validation studies of the Distress Thermometer worldwide^21^ proposed an alternative pooled score of ⩾4. A subsequent meta-analysis with subgroup analysis recommended an optimal cut-off ⩾6 for patients with cancer at the end of life,^22^ however pointed out the inadequate specificity ( < 0.60) of pooled scores at cut-offs of ⩾4 and ⩾5 from a limited number of available studies.^23^, ^24^

Despite recommendation for further validation work there has been little progress since publication of the meta-analyses,^22^ with only four validation studies in total of the Distress Thermometer in palliative care settings.^25,26^ The two studies published since the meta-analysis^22^ propose an optimal clinical cut-off of ⩾5 and report high sensitivity but poor specificity (i.e. <  60%). There is acknowledged variation in Distress Thermometer clinical cut-offs according to instrument language, country, clinical population and setting, and therefore a need for revalidation in new populations.^21^ A potential limitation of existing validation studies is that three^23,24,26^ have derived data from either heterogeneous clinical populations of both patients with malignant and non-malignant disease^24,26^ or across settings, for example, acute hospital and inpatient hospice units.^23^ The other existing study^25^ included a more homogenous sample of patients with advanced cancer with pain but in an acute hospital setting. Specialist palliative care settings may include hospital, home, hospice inpatient units, outpatients and day services.^27^ On the basis of the existing evidence, there is no clarity on which clinical cut-off is optimal for implementation of the Distress Thermometer with patients with advanced cancer in a hospice setting (inpatient or day hospice).

The current study provides the first validation of the Distress Thermometer in patients with advanced cancer receiving specialist palliative care in a hospice setting (inpatient & day hospice). The study aims to:

- Evaluate the sensitivity and specificity of the Distress Thermometer in screening for distress, anxiety and depression, using the Hospital Anxiety and Depression Scale^28^ as a reference measure
- Identify the optimal cut-off points for the Distress Thermometer at which to make referrals for further psychological assessment
- Identify socio-demographic and clinical factors which are associated with heightened psychological morbidity among this population

## Method

### Description of the data and the population

A secondary analysis of data held by a UK hospice was undertaken. The hospice services include an 18-bed inpatient unit, and a day hospice with 5 clinics per week. The data were previously collected during the course of routine care to inform a psychological needs assessment. The psychological needs assessment database contained data on patients consecutively admitted to day hospice or the inpatient unit between September 2014 and August 2016. The Distress Thermometer^19^ and the Hospital Anxiety and Depression Scale^28^ were administered upon patient admission, and completed independently by the patient, or with clinician support if needed. Both measures were administered consecutively (Hospital Anxiety and Depression Scale followed by Distress Thermometer) by palliative care physicians who were unaware of the patient’s score on the reference test (Hospital Anxiety and Depression Scale) which was calculated at later date. For the patients who were approached for screening, demographic and clinical data were collected alongside reasons for non-completion where appropriate. For the purposes of the current study, data of all patients were eligible for inclusion in the secondary analysis, with the exception of data from patients with non-malignant disease.

### Measures

#### Socio-demographic and clinical characteristics

Data included disease malignancy, International Classification of Diseases-10 disease classification- neoplasms,^29^ locally advanced/metastatic disease, Eastern Cooperative Oncology Group performance status,^30^ previous mental health condition (yes/no) and if currently prescribed specific medication (opiates, anxiolytics, anti-depressants, hypnotics, anti-psychotics, anti-epileptics). Socio-demographic data included age, gender, ethnicity, and marital status.

#### Screening Measures

The Distress Thermometer Version 2^19^ is the index test. The Distress Thermometer is a single-item, 11-point visual analogue scale, with respondents indicating how distressed they have felt over the past week (from “No Distress” to “Extreme Distress”).

The Hospital Anxiety and Depression Scale^28^ is the reference measure. The Hospital Anxiety and Depression Scale is a 14-item questionnaire for physically ill patients with two subscales; anxiety (Hospital Anxiety and Depression Scale-Anxiety;7-items) and depression (Hospital Anxiety and Depression Scale-Depression;7-items) with each item rated on a 0 to 3 scale. Each subscale has a total score ranging from 0 to 21, with a higher total score indicating a higher level of anxiety or depression. A total overall score for distress [Hospital Anxiety and Depression Scale-Total] ranges from 0 to 42. Guidelines suggest clinical caseness of ⩾8 on each subscale, and ⩾15 for total score.^31^ The Hospital Anxiety and Depression Scale is the most frequently used mood scale in cancer and palliative settings,^32^ and the most frequently used reference measure for validation of the Distress Thermometer in cancer populations.^21^

### Analysis

Statistical analysis was performed using the Statistical Package for Social Sciences (version 22.0). Demographic and clinical data were analyzed using descriptive statistics. The relationship between demographic/clinical characteristics and distress was investigated with correlations, independent *t-*tests, and analysis of variance. Receiver operating characteristics analysis was used to compare the three recommended cut-off points of the Distress Thermometer (4, 5 & 6)^20–22^ to the Hospital Anxiety and Depression Scale cut-off scores of ⩾8 on each subscale, and ⩾15 for total score.^32^ The optimal cut-off score was determined according to the point at the top left of the curve. The sensitivity, specificity, positive predictive value, negative predictive value and positive and negative likelihood ratios were calculated for each cut-off of the Distress Thermometer score against the Hospital Anxiety and Depression Scale.

### Sample size calculation

A medium effect size is equivalent to an area under the curve in receiver operating characteristics analysis of 0.639.^33^ A sample size of 138 is sufficient to detect an area under the curve of 0.639 with 90% power, using an alpha value of 0.05.

### Ethics

National Health Service research ethics approval for the secondary analysis was obtained from Office for Research Ethics Committees Northern Ireland (REC reference: 17/NI/0036) and research governance approval from the Marie Curie Hospice Belfast Research Governance Committee.

## Results

All patients were offered the measures to complete upon admission, with the exception of those patients who were unconscious at the time of admission, had rapidly deteriorating health, fatigue, agitation or confusion (*n* = 141 inpatient unit). A total of 202 patients were approached for screening. Five patients declined to participate and another 29 were not able to participate primarily because they were too unwell. Of the remaining 168 patients, 139 provided complete information on the Hospital Anxiety and Depression Scale and Distress Thermometer. Participants had a mean (standard deviation) age of 67.26 (11.72) years. Table 1 shows the other medical and demographic information for this sample.

**Table 1.** Characteristics of hospice patients (*n* = 139).

	*N* (%)
**Gender**	
Male	53 (38)
Female	86 (62)
**Ethnicity**	
White	136 (98)
Other	3 (2)
**Marital Status**	
Never Married	16 (12)
Divorced	18 (13)
Married/Long Term Partner	74 (53)
Widowed	31 (22)
**Locally Advanced/Metastatic**	
Locally Advanced	44 (32)
Metastatic	92 (67)
Haematological	1 (0.7)
Missing data	2 (1.4)
**Eastern Cooperative Oncology Group performance status**	
1	50 (37)
2	31 (23)
3	43 (32)
4	12 (9)
Missing data	3 (2.2)
Previous mental health issues (yes/no)	59/139 (42)
**Current Medication**	
Opiates	108 (78)
Anxiolytics	33 (24)
Anti Depressants	43 (31)
Hypnotics	20 (14)
Anti Psychotics	14 (10)
Anti Epileptics	30 (22)
**Cancer Site**	
Respiratory & intrathoracic	40 (29)
Digestive organs	31 (22)
Breast	19 (14)
Urinary tract	12 (9)
Female genital organs	7 (5)
Male genital organs	6 (4)
Lip, oral cavity & pharynx	6 (4)
Other	18 (13)

### Distress thermometer and hospital anxiety and depression scale descriptive statistics

Scores on the Distress Thermometer ranged from 0 to 10, with a mean score of 5.40 (standard deviation = 2.91). The total Hospital Anxiety and Depression Scale score ranged from 2 to 34, with a mean score of 17.35 (standard deviation = 8.31). Mean (standard deviation) scores for the anxiety and depression subscales of the Hospital Anxiety and Depression Scale were 8.55 (4.76) and 8.80 (4.69) respectively. The number of participants experiencing clinically significant levels of anxiety, depression and overall distress according to the Hospital Anxiety and Depression Scale were 79/139 (43%), 86/139 (62%) and 87/139 (63%), respectively.

### Receiver operating characteristic analysis and optimal cut-off score

The scores from the Distress Thermometer were compared to Hospital Anxiety and Depression Scale total, anxiety, and depression scores, using receiver operating characteristics analysis (see Table 2).

**Table 2.** Results of a receiver operating characteristics analysis for distress thermometer cut-off scores of 4 to 6 compared with the hospital anxiety and depression scale.

	Proposed cut-off on distress thermometer	Area under the curve (95% CI)	Sensitivity	Specificity	Positive predictive value	Negative predictive value	Positive likelihood ratio	Negative likelihood ratio
**Hospital Anxiety and Depression Scale-Total ⩾15**	4	0.696 (0.601–0.792)	0.874	0.519	0.752	0.711	1.82	0.24
**Hospital Anxiety and Depression Scale-Total ⩾15**	5	0.698 (0.605–0.792)	0.782	0.615	0.773	0.627	2.03	0.35
**Hospital Anxiety and Depression Scale-Total ⩾15**	6	0.699 (0.608–0.789)	0.667	0.731	0.806	0.567	2.48	0.46
**Hospital Anxiety and Depression Scale-Anxiety ⩾8**	4	0.729 (0.640–0.818)	0.924	0.533	0.598	0.894	1.98	0.14
**Hospital Anxiety and Depression Scale- Anxiety ⩾8**	5	0.720 (0.631–0.808)	0.823	0.617	0.620	0.817	2.15	0.29
**Hospital Anxiety and Depression Scale- Anxiety ⩾8**	6	0.736 (0.650–0.821)	0.722	0.750	0.683	0.776	2.89	0.37
**Hospital Anxiety and Depression Scale- Depression ⩾8**	4	0.676 (0.579–0.772)	0.860	0.491	0.733	0.684	1.69	0.29
**Hospital Anxiety and Depression Scale- Depression ⩾8**	5	0.661 (0.566–0.756)	0.756	0.566	0.739	0.588	1.74	0.43
**Hospital Anxiety and Depression Scale- Depression ⩾8**	6	0.675 (0.582–0.767)	0.651	0.698	0.778	0.552	2.16	0.50

**Figure 1.**
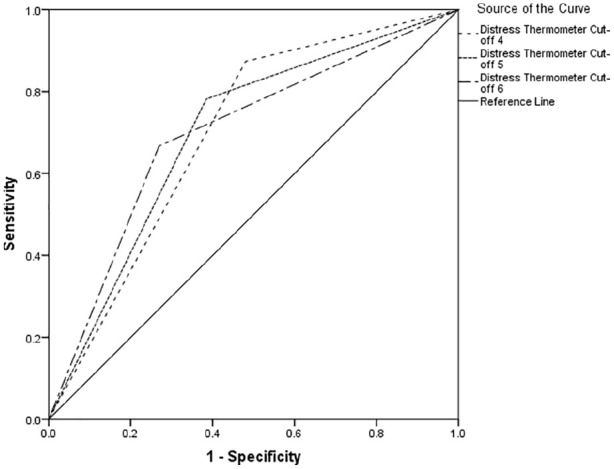
Receiver operating characteristics curve for Hospital Anxiety and Depression Scale-Total Score.

**Figure 2.**
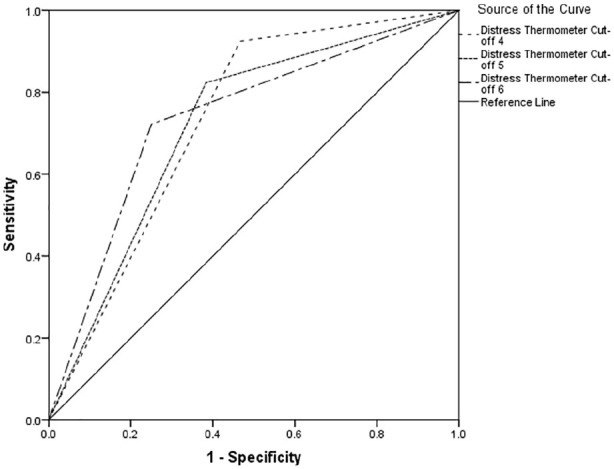
Receiver operating characteristics curve for Hospital Anxiety and Depression Scale-Anxiety Score.

**Figure 3.**
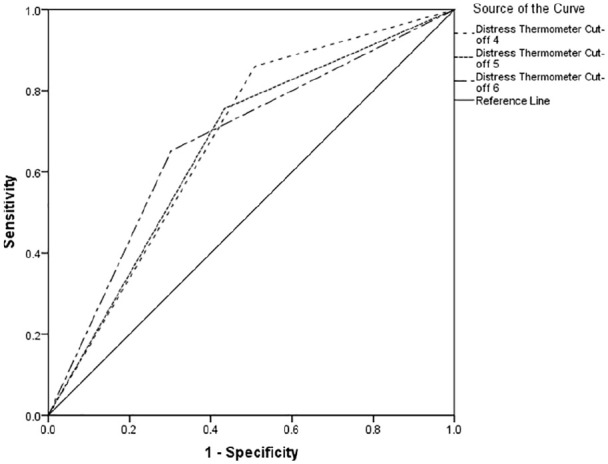
Receiver operating characteristics curve for Hospital Anxiety and Depression Scale-Depression Score.

Table 2 indicates between 66% and 74% of cases of clinically significant psychological morbidity would be correctly identified for referral for further assessment. Using recommended guidelines,^34^ the overall performance reports poor to fair discrimination across all cut-offs (see Figures 1–3).

The area under the curve was optimal with a Distress Thermometer cut-off ⩾6 for distress, with a Distress Thermometer cut-off ⩾6 for anxiety and a Distress Thermometer cut-off ⩾4 for depression. However, there is little difference between the area under the curve scores for the different Distress Thermometer cut-off points. Table 3 reports cross tabulation of the index test results (Distress Thermometer) by the results of the reference standard (Hospital Anxiety and Depression Scale-Depression, Hospital Anxiety and Depression Scale-Anxiety & Hospital Anxiety and Depression Scale-Total Score).

**Table 3.** Frequency of correct and incorrect classifications when using the Distress Thermometer cutoff ⩾5, with the Hospital Anxiety and Depression Scale cutoffs as reference standard.

	Index test (Distress Thermometer) cut-off score	
**Reference test (Hospital Anxiety and Depression Scale) cutoff score**	Below distress thermometer cut-off ⩾5 *N* (%)	Above distress thermometer cut-off ⩾5 *N* (%)
Below Hospital Anxiety and Depression Scale-Depression⩾8 *N* (%)	30 (22%)	23 (17%)
Above Hospital Anxiety and Depression Scale-Depression ⩾8 *N* (%)	21 (15%)	65 (47%)
Below Hospital Anxiety and Depression Scale-Anxiety⩾8 *N* (%)	37 (27%)	23 (17%)
Above Hospital Anxiety and Depression Scale-Anxiety⩾8	14 (10%)	65 (47%)
*N* (%)		
Below Hospital Anxiety and Depression Scale-Total⩾15 *N* (%)	32 (23%)	20 (14%)
Above Hospital Anxiety and Depression Scale-Total⩾15 *N* (%)	19 (14%)	68 (49%)

### Factors influencing the distress thermometer and hospital anxiety and depression scale

There was a weak, negative correlation found between age and the Hospital Anxiety and Depression Scale-Anxiety (*r* = −0.19, *p* = 0.027), the Hospital Anxiety and Depression (−0.19, *p = *0.026), and the Distress Thermometer (*r* = −0.27, *p* = 0.001). Associations with the other demographic and clinical variables are reported in Table 4. There was a moderate-large effect on Hospital Anxiety and Depression Scale-Anxiety, Hospital Anxiety and Depression Scale–Depression, Hospital Anxiety and Depression Scale-Total and the Distress Thermometer among respondents with previous mental health issues and those who have been prescribed anxiolytics or anti-depressants. Females scored moderately higher on the Hospital Anxiety and Depression Scale-Anxiety and the Hospital Anxiety and Depression Scale-Total and those prescribed hypnotics scored moderately higher on the Distress Thermometer. Those who were divorced scored moderately higher on the Hospital Anxiety and Depression Scale-Depression and Hospital Anxiety and Depression Scale-Total; and those prescribed opiates or with a performance status score of 4 scored moderately higher on the Hospital Anxiety and Depression Scale-Depression.

**Table 4.** One-way ANOVA of associations between demographic and clinical variables and distress thermometer/hospital anxiety and depression scale scores.

	Distress thermometer	Hospital anxiety and depression scale- anxiety	Hospital anxiety and depression scale- depression	Hospital anxiety and depression scale-total
Gender	*F*(1,137) = 3.15, *p* = 0.078	*F*(1,137) = 13.04, *p* < 0.001*	*F*(1,137) = 1.84, *p* = 0.177	*F*(1,137) = 7.93, *p* = 0.006*
Marital status	*F*(3,135) = 1.77, *p* = 0.156	*F*(3,135) = 1.78, *p* = 0.155	*F*(3,135) = 3.02, *p* = 0.032*	*F*(3,135) = 3.06, *p* = 0.030*
Locally advanced vs metastatic	*F*(1,137) = 1.74, *p* = 0.189	*F*(1,137) = 1.21, *p* = 0.274	*F*(1,137) = 2.59, *p* = 0.109	*F*(1,137) = 2.38, *p* = 0.125
Performance status	*F*(3,132) = 1.51, *p* = 0.216	*F*(3,132) = 0.66, *p* = 0.580	*F*(3,132) = 4.29, *p* = 0.006*	*F*(3,132) = 2.48, *p* = 0.064
Previous mental health issues	*F*(1,137) = 21.70, *p* < 0.001*	*F*(1,137) = 33.47, *p* < 0.001*	*F*(1,137) = 16.02, *p* < 0.001*	*F*(1,137) = 32.25, *p* < 0.001*
Cancer site	*F*(7,131) = 1.12, *p* = 0.353	*F*(7,131) = 2.07, *p* = 0.051	*F*(7,131) = 0.71, *p* = 0.664	*F*(7,131) = 0.53, *p* = 0.811
Medication-				
Opiates	*F*(1,137) = 0.08, *p* = 0.780	*F*(1,137) = 0.12, *p* = 0.728	*F*(1,137) = 6.31, *p* = 0.013*	*F*(1,137) = 2.57, *p* = 0.112
Anxiolytics	*F*(1,137) = 9.50, *p* = 0.002*	*F*(1,137) = 16.02, *p* < 0.001*	*F*(1,137) = 19.53, *p* < 0.001*	*F*(1,137) = 23.81, *p* < 0.001*
Anti-Depressants	*F*(1,137) = 6.33, *p* = 0.013*	*F*(1,137) = 11.62, *p* = 0.001*	*F*(1,137) = 5.06, *p* = 0.026*	*F*(1,137) = 10.49, *p* = 0.002*
Hypnotics	*F*(1,137) = 4.76, *p* = 0.031*	*F*(1,137) = 3.58, *p* = 0.061	*F*(1,137) = 2.42, *p* = 0.122	*F*(1,137) = 3.87, *p* = 0.051
Anti-psychotics	*F*(1,137) = 0.51, *p* = 0.478	*F*(1,137) = 0.08, *p* = 0.779	*F*(1,137) = 0.17, *p* = 0.684	*F*(1,137) = 0.01, *p* = 0.944
Anti-Epileptics	*F*(1,137) = 1.27, *p* = 0.262	*F*(1,137) = 0.63, *p* = 0.428	*F*(1,137) = 0.56, *p* = 0.456	*F*(1,137) = 0.77, *p* = 0.381

## Discussion

This study provides the first validation of the Distress Thermometer in an advanced cancer population receiving specialist palliative care in a day or inpatient hospice setting. This adds to the small number of studies^23–26^ suggesting the Distress Thermometer to be a valid method for screening for indicative psychological morbidity in palliative care.

The optimal Distress Thermometer cutoff according to the area under the curve when screening for distress and anxiety is proposed as ⩾6 for distress and anxiety and as ⩾4 for depression in the current study. It must however be noted that there is little difference between the area under the curve score for Distress Thermometer cut-offs ⩾4, ⩾5 and ⩾6, yet Distress Thermometer cut-offs, ⩾5 and ⩾6 offer the smallest range of confidence interval. It is therefore the decision of the authors to amend the test decision criterion to alter the balance between sensitivity and specificity towards developing an optimal screening test. In their systematic review,^22^ Ma and colleagues argue for a Distress Thermometer cut-off ⩾6 to increase the specificity of the measure with a trade-off in relation to sensitivity. We recommend the utility of the Distress Thermometer as a distress screening measure be prioritized, which places emphasis on the ability of the tool to identify those without the disorder with minimal false negatives. On this basis we believe a Distress Thermometer cut-off of ⩾5 to be optimal in screening for distress (Sensitivity-0.78, Specificity-0.62), for anxiety (Sensivity-0.82, Specificity-0.62) and for depression (Sensitivity = 0.76, Specificity-0.57). This ensures that sensitivity is prioritized above specificity (reported as fair to good), while guaranteeing specificity to be at a level higher than chance (reported as poor to fair). With a cut-off of ⩾5 the Distress Thermometer reports poor case-finding ability however the cost of false positives is likely to be material; time/cost on further psychological assessment. A Distress Thermometer cut-off of ⩾5 is in line with the existing research in palliative care.^23-26^ Across all cut-offs the specificity is poor to fair, which may be as the Distress Thermometer was developed as a screen for multi-factorial distress rather than simply clinical mood disorders. It may be that rather than the Distress Thermometer detecting anxiety or depression, it is detecting variance shared with general distress. It is important to consider the performance of the measure within this broader context.

There has been limited validation of unidimensional measures for complex psychological constructs,^18^ despite evidence that clinicians prefer slightly less accurate but briefer screening measures^35^ suggesting that the Distress Thermometer may be acceptable in practice. The accuracy reported of the Distress Thermometer in the current study is in line with other ultra-short distress screening measures,^18^ the existing Distress Thermometer validation studies in palliative care^23–26^ and the broader Distress Thermometer validation research in oncology.^21^ Using any Distress Thermometer cut-off, the sensitivity of the Distress Thermometer is poor to good, however the specificity is poor to fair. The Distress Thermometer is good at identifying psychological morbidity, but poor at identifying individuals without psychological morbidity with a high degree of false positives. This contributes towards an area under the curve which is poor to fair. It must be considered however that in mental health research where the index test is unlikely to be perfect, it is impossible for the area under the curve to reach 1.00.^36^ When questionnaires produce an area under the curve greater than 0.90, it is more likely to indicate design flaws.^37^

### Strengths and limitations

The current study has a number of strengths. The sample size (*n = *139) is relatively large for a population receiving specialist palliative care. This is the first validation study specifically with a hospice population (inpatient and day hospice) and therefore provides accurate clinical cut-offs for use of the Distress Thermometer as a psychological screening tool in this setting. Importantly, the current study derived data only from patients with advanced cancer, as with only two other studies internationally.^23,25^ This is an improvement from a significant number of validation studies deriving data from patients at various stages of the cancer trajectory.^21^

There are several limitations to the current study. Firstly, the Hospital Anxiety and Depression Scale^28^ was used as the reference test rather than the Structured Clinical Interview for Diagnostic and Statistical Manual of Mental Disorders –V.^5,38^ However, with one exception^23^ the Structured Clinical Interview for Diagnostic and Statistical Manual of Mental Disorders is rarely used as the reference measure for validation of the Distress Thermometer. The Hospital Anxiety and Depression Scale is the dominant reference test in validation studies of the Distress Thermometer.^21^ It must however be acknowledged that there is uncertainty with the latent structure of the measure^39^ in addition to the content validity.^40^ Authors have proposed the Hospital Anxiety and Depression Scale best fits a bifactor structure and suffers from saturation of a general distress factor, meaning there are issues distinguishing between anxiety and depression.^39^ This therefore results in more confidence in the use of the Hospital Anxiety and Depression Scale as a reference test for distress rather than for anxiety or depression. Lastly, with acknowledgement that the Distress Thermometer needs to be revalidated in different cultures^21^ the generalizability of these findings outside of the UK/Ireland warrants caution. However, our proposed Distress Thermometer cut-off ⩾5 aligns to recommendations based upon Chinese^25^ and German^26^ palliative care samples with similar performance in relation to sensitivity and specificity which may suggest that clinical characteristics and setting are more important determinants.

### Implications for practice

There is evidence that the majority of palliative care providers do not use a validated tool to screen for psychological morbidity.^16,17^ Clinical guidelines for distress management in cancer populations recommend screening for distress in all patients, followed by clinical diagnostic interview for those who screen positive.^41–43^ Palliative care guidelines^44^ are consistent with this recommendation but do not identify specific examples of tools to be used, or the timing of administration, instead emphasizing, “whenever possible and appropriate, a validated and context-specific assessment tool is used;”^44^ p64]. The current study administered the Distress Thermometer and Hospital Anxiety and Depression Scale on admission to hospice, identifying a significant proportion of patients experiencing clinically significant levels of anxiety, depression and overall distress. This provides evidence of the utility of screening at this early stage within the hospice setting. The findings suggest that particular attention should be given to patients, who are younger, female, have previous mental health issues and who have been prescribed anxiolytics, antidepressants, opiates and hypnotics. It is also worth noting the recommendation that tools with acceptable sensitivity among patients with high symptom burden are particularly needed.^17^ The Distress Thermometer as a one-item measure is quick to administer and unlike other tools used within this setting^45^ does not rely on aspects of psychological morbidity that are also common somatic symptoms of illness.

### Future research

There is some evidence that the accuracy of the Distress Thermometer can be improved with the addition of three emotion thermometers (depression, anxiety, anger) and one outcome thermometer (need for help).^46,47^ As far as the authors are aware the addition of emotion thermometers has not been validated in a palliative care setting, and hence this is an important area for future research. There is evidence in cancer settings demonstrating patient acceptability of a five-step process integrating the Distress Thermometer with patient review, need for help and referral information.^48^ Further research is needed to ensure use of the Distress Thermometer is integrated within an evidence-based pathway for identification and management of distress.^16,17^

## Conclusion

In conclusion, findings suggest that the Distress Thermometer is a valid, ultra-short screening measure for use with advanced cancer patients receiving specialist palliative care in the inpatient or day hospice setting. It is recommended that specialist palliative care clinicians implementing the Distress Thermometer in this setting should use a cut-off of ⩾5 when screening for anxiety, depression or distress. As the specificity of the measure is poor, service providers should be aware of potential for significant false positives and ensure the Distress Thermometer is integrated within an evidence-based pathway which includes further psychological assessment.

## Supplemental Material

Distress_Thermometer_Supplementary_File – Supplemental material for Validation of the Distress Thermometer in patients with advanced cancer receiving specialist palliative care in a hospice setting Click here for additional data file. Supplemental material, Distress_Thermometer_Supplementary_File for Validation of the Distress Thermometer in patients with advanced cancer receiving specialist palliative care in a hospice setting by Lisa Graham-Wisener, Martin Dempster, Aaroon Sadler, Luke McCann and Noleen K McCorry in Palliative Medicine
