# Supplementary figures and images for: Validation of the Distress Thermometer in patients with advanced cancer receiving specialist palliative care in a hospice setting
Source: Palliat Med. 2020 Sep 11;35(1):120–9. doi: 10.1177/0269216320954339 (PMC7797615; doi:10.1177/0269216320954339)

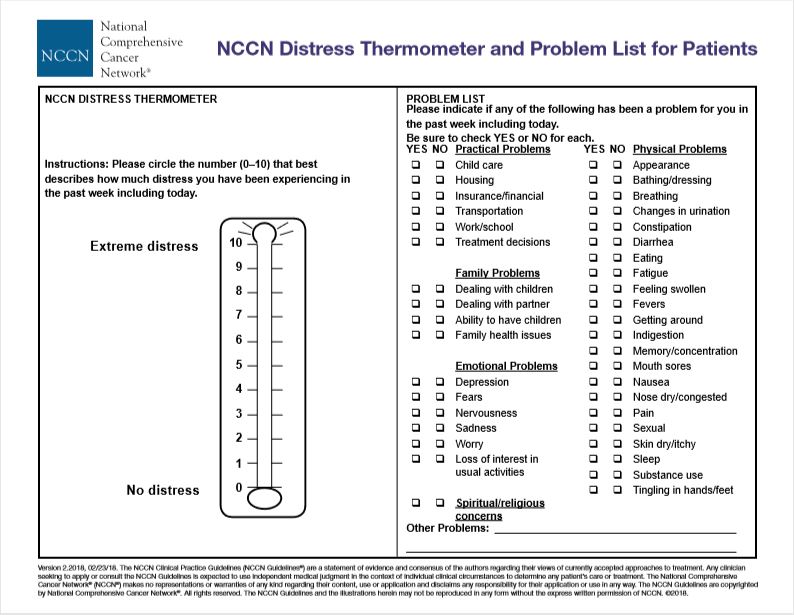

Supplement: Distress_Thermometer_Supplementary_File – Supplemental material for Validation of the Distress Thermometer in patients with advanced cancer receiving specialist palliative care in a hospice setting [file Distress_Thermometer_Supplementary_File.JPG]
